# LDH Nanocubes Synthesized with Zeolite Templates and Their High Performance as Adsorbents

**DOI:** 10.3390/nano11123315

**Published:** 2021-12-07

**Authors:** Moftah Essa Elkartehi, Rehab Mahmoud, Nabila Shehata, Ahmed Farghali, Shimaa Gamil, Amal Zaher

**Affiliations:** 1 Department of Environmental Science and Industrial Development, Faculty of Postgraduate Studies for Advanced Sciences, Beni-Suef University, Beni-Suef 62511, Egypt; Moftah1@yahoo.com (M.E.E.); nabila.shehata@yahoo.com (N.S.); 2 Department of Chemistry, Faculty of Science, Beni-Suef University, Beni-Suef 62511, Egypt; 3 Materials Science and Nanotechnology Department, Faculty of Postgraduate Studies for Advanced Sciences, Beni-Suef University, Beni-Suef 62511, Egypt; d_farghali@yahoo.com (A.F.); osha_gemy2005@yahoo.com (S.G.)

**Keywords:** wastewater, layered double hydroxide (LDH), methylene blue, isotherm, kinetic models

## Abstract

In this work, the efficiency of the adsorptive removal of the organic cationic dye methylene blue (MB) from polluted water was examined using three materials: natural clay (zeolite), Zn-Fe layered double hydroxide (LDH), and zeolite/LDH composite. These materials were characterized via X-ray diffraction (XRD), Fourier transform infrared (FTIR) spectroscopy, high-resolution transmission electron microscopy (HRTEM), energy dispersive X-ray (EDX) diffraction (XRF), low-temperature N_2_ adsorption, pore volume and average pore size distribution and field emission scanning electron microscopy (FE-SEM). The properties of the applied nanomaterials regarding the adsorption of MB were investigated by determining various experimental parameters, such as the contact time, initial dye concentration, and solution pH. In addition, the adsorption isotherm model was estimated using the Langmuir, Freundlich, and Langmuir–Freundlich isotherm models. The Langmuir model was the best-fitting for all applied nanomaterials. In addition, the kinetics were analyzed by using pseudo-first-order, pseudo-second-order, and intraparticle diffusion models, and the pseudo-second-order model was an apparent fit for all three applied nanomaterials. The maximum Adsorption capacity toward MB obtained from the materials was in the order zeolite/LDH composite > zeolites > Zn-Fe LDH. Thus, the zeolite/LDH composite is an excellent adsorbent for the removal of MB from polluted water.

## 1. Introduction

In recent years, large quantities of harmful dyes have been used in various industries, such as in pharmaceutical products, textiles, leather, cosmetics, food, and paints [1]. According to recent studies, more than 10,000 types of organic dyes used in industry are discharged as effluents into the environment, causing harmful pollution [2,3,4]. Synthetic dyes are difficult to biodegrade and cause severe public health concerns, even at low concentrations [5]. Therefore, alternative techniques for removing dyes from wastewater are needed to decrease pollutant concentrations to acceptable levels.

Dyes are classified according to their nuclear structures into anionic, cationic, and nonionic categories [6]. Among the various cationic dye types, methylene blue (MB) is a notable candidate mainly used in the textile and printing industries [7]. Continuous exposure to MB contamination in wastewater can cause harmful effects such as shock, vomiting, and increased heart rate. In line with green industry principles, the removal of MB dye has become a serious research subject. Various techniques have been developed for the removal of pollutants from wastewater, e.g., photocatalytic degradation [8], membrane filtration [9], electrochemical methods, advanced oxidation [10], and adsorption. The adsorption technique has received much attention compared to other techniques because of its high efficiency, low cost, ease of use, and the availability of numerous adsorbents. Another aspect favoring the use of adsorption techniques is the elimination of secondary pollutant formation, which can occur through the other techniques [11]. Recently, great efforts have been devoted to the identification of low-cost and more efficient natural and synthetic materials as new adsorbents for dye removal from wastewater.

In our research, we applied three nanomaterials as adsorbents for MB removal and estimated the most effective nanomaterial for the adsorption process. The first applied material was a layered double hydroxide (LDHs), which belongs to a class of two-dimensional (2D) nanostructured materials previously known as ionic lamellar compounds. The lamellar structure of LDHs is favored as a catalyst for different practical applications due to its tunable chemical composition, unique structure, nontoxicity, and wide variety of material properties [12,13]. These characteristics allow them to be used as adsorbents [14], and in catalysts [15], fuel cells [16], drug delivery [17], CO_2_ capture [18], and many other potential applications. In particular, LDHs exhibit effective performance as an adsorbent for organic pollutants, mainly due to their low cost, positively charged layers with anion exchange capability, colloidal and thermal behavior, and high surface area [19,20]. Various functionalization strategies have been applied to LDHs to achieve high performance in various applications [19,21]. The second applied material was a natural zeolite, it could be defined as a porous crystalline inorganic polymer containing SiO_4_ and AlO_4_ tetrahedra (alumino-silicate) with three-dimensional structure, this structure being filled with water molecules and ions, having great freedom of movement. zeolites have received considerable attention [22,23]. In particular it was of the clinoptilolite type (Figure S1) [24]. It has been used for various environmental applications, such as catalysis and water purification [25,26]. This is due to its negative charge resulting from the replacement of Al^+3^ by Si^+4^; this negative charge is neutralized by exchangable cations [21]. On the other hand, it has remarkable adsorption power, attributed to its high surface area and porous nature [27]. Recently, more attention has been focused on the surface modification of different nanoparticles using zeolites [27]. Several authors have used zeolites for the production of several promising polymer nanocomposites for wastewater purification [28,29]. They found that zeolite-modified composites are very promising in the removal of toxic metals and could therefore act as potential adsorbents for water-treatment processes, exhibiting superior adsorption [22]. Many researchers have modified zeolites with magnetic nanoparticles as adsorbents for MB removal from aqueous solution [23,30].

Moreover, modification with a homogeneous support can improve LDH performance, thermal stability, morphology, and lifetime by offering the opportunity to support the homogeneous reaction over a hybridized LDH. Nowadays, various supporting materials such as chitosan, activated carbon, and zeolite, have been utilized in the generation of LDH-based hybrids. However, zeolite is the most attractive alternative for environmental applications. Novel Zn-LDH-hybridized zeolites were investigated for the removal of phosphorus. The results showed that the individual three-dimensional frameworks of the Zn-LDH nanocomposite are important for improving the adsorption process in wastewater treatment applications [31] (Table 1).

The third material applied in this study was a zeolite/LDH composite. To our knowledge, no previous study on the binary effect of zeolite and LDH has been undertaken. Therefore, we selected zeolite/LDH composite as an efficient, low-cost, bifunctional adsorbent that is promising for MB removal from wastewater. Our study shows that the hybridization of multidimensional nanomaterial creates new composites featuring the advantages of each component, which is a promising method for the production of multifunctional adsorbents with unusual properties [12], in addition to evaluating the MB removal performance and determining the optimal conditions of use.

## 2. Experimental Details

### 2.1. Materials

Zn(NO_3_)_2_.6H_2_O was purchased from Chem-Lab NV (Zedelgem, Belgium), Fe(NO_3_)_3_.9H_2_O. Hydrochloric acid was supplied by Carlo Erba Reagents (Ain St, Cairo, Egypt) while NaOH was obtained from Piochem Laboratory Chemicals, (Giza, Egypt). MB powder was purchased from Oxford Laboratory Reagents (Hyderabad, India). Commercial zeolite with a particle size in the range of 1–10 µm was used for the base particles; all mentioned chemicals were used as received without any additional purification. The experiments and preparation of the materials were performed using deionized water free of CO_2._

### 2.2. Methods

#### 2.2.1. Synthesis of Zn-Fe LDH

**Figure 1:**
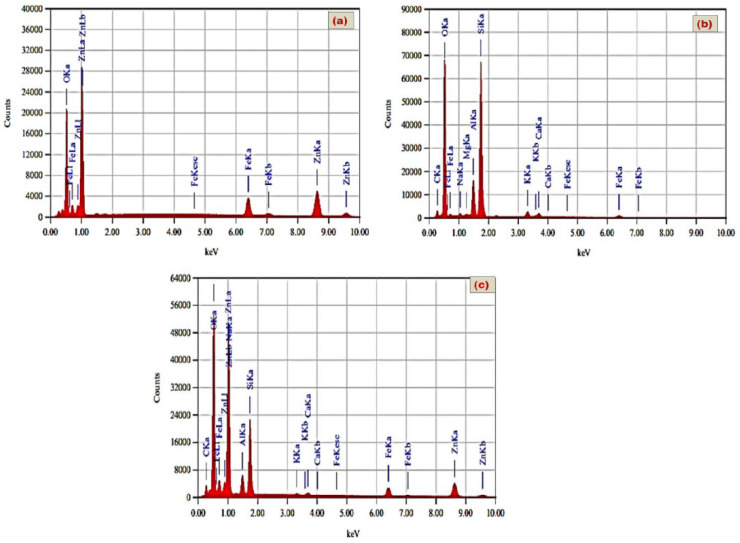
EDX of the prepared Zn-Fe LDH (**a**); zeolite (**b**) and zeolite/Zn-Fe LDH nanocomposite(**c**).

A co-precipitation method was used to prepare Zn–Fe LDH. Solutions of zinc and iron as nitrate precursors were added together in a 4:1 molar ratio (Figure 1a). NaOH (2.0 M) solution was added dropwise at 0.10 mL/min until pH 10 to achieve complete precipitation. The resulting material was aged and kept for 20 h at 60 ± 0.5 °C; after that, the obtained product was filtered and washed several times using distilled water to dispose of excess OH^−^, then washed with ethanol. Finally, the resulting adsorbent sample was dried for 12 h at 60 ± 0.5 °C [36].

#### 2.2.2. Preparation of Zeolite

The commercial zeolite was processed in a photon ball milling vessel under the conditions described in Table 2, for 10 h, under a continuous mechanical rotation speed of 200 rpm. The elemental analysis results of zeolite using the EDX and XRF techniques is presented in Figure 1b and Figure S1.

#### 2.2.3. Preparation of Zeolite/Zn-Fe LDH Composite

The zeolite/Zn-Fe LDH composite (with a Zn/Fe/zeolite molar ratio of 4:1:0.5) (Figure 1c) was prepared by repeating the above procedure and adding a solution of zeolite (1.5 g of zeolite dissolved in 50 mL bidistilled water) to the aqueous medium before precipitation at pH 10.0 using 2.0 mol L^−1^ NaOH solution. The suspension of zeolite/LDH precipitate was stirred for 20 h at room temperature, and then filtered. After that, the formed nanocomposites were collected and washed using bidistilled water and ethanol several times and finally dried for 12 h at 60 ± 0.5 °C.

### 2.3. Characterizations of the Prepared Materials

The formed zeolite, LDH, and zeolite/LDH composite were characterized by XRD (PANalytical Empyean, Uppsala, Sweden). An accelerating voltage of 40 KV was applied, with the scan angle ranging from 5 to 60°, a scan step of 0.05°, and a 30 mA current. A Vertex 70 FTIR-FT Raman instrument (Bruker, Thane, Maharashtra, India) was used to determine the vibration of the chemical bonds. The frequency range of 400–4000 cm^−1^ was applied, using a potassium bromide disc. The morphology of the materials was estimated using a field emission scanning electron microscope (FE-SEM, SU7000, Hitachi -High, Mannheim, Germany) EDX (Quanta FEG250, Berlin, Germany) was applied to determine the molar ratio in the prepared samples. The BET specific surface area, pore size distribution, and pore volume of the nano-adsorbents were estimated by N_2_ adsorption–desorption method by an automatic surface analyzer (TriStar II 3020, Micrometrics, Norcross, Georgia, USA). High-resolution transmission electron microscopy (HRTEM, JEM 2100, JEOL, Welwyn Garden, Hertfordshire AL7 1LT, U.K.) was applied to determine the microstructures of the produced materials. XRF analysis was performed to the natural zeolite to confirm the structure using a XRF-ARL-9900 system (olympus, Hamburg, Germany). The sample preparation procedure of the zeta potential measurements was as explained in a previous study [37].

### 2.4. Adsorption Arrays

Several experiments were conducted to obtain data about the effects of the solution pH, initial dye concentration, adsorbent amount, and contact time on the dye adsorption. All experiments were carried out in batch operating systems at ambient temperature. Beginning with a standard stock aqueous solution of MB with an initial concentration of 200 mg/L, a series of diluted solutions was prepared to obtain the calibration curve (10–80 mg/L). Falcon tubes (50 mL) were prepared by adding 0.05 g of the synthesized sorbent and 20 mg/L of dye as a pollutant, then we adjusted the pH of the dye tubes to 3, 5, 7, 9, or 10 using 0.1 N HCl or 0.1 N NaOH and applying a pH meter (751 Titrino, Metrohm, Switzerland). All experiments were conducted in darkness, and the Falcon tubes were put on an orbital shaker (SCILOGEX SK- O330- Pro, SCILOGEX, Alabama, USA) at 200 rpm for 20 h, until equilibrium was reached, to estimate the residual concentration of MB at a wavelength of 660 nm [38] using a UV–visible spectrophotometer (UV-2600, Shimadzu, Tokyo, Japan). To check the reproducibility of experiments, all experiments were performed in triplicate. The influence of the initial dye concentration was also examined for each dye by mixing the optimum dose of sorbent with 50 mL of the dye solution at the optimum pH. The adsorption experiments were conducted in batch mode to estimate the effects of the initial concentration of MB. The amount of dye removed was estimated using the following equations:

(1) $$q_{e}=\left(C_{o}-C_{t}\right)*V/W$$

(2) $$Q\%=\left(C_{o}-C_{t}\right)*100/C_{o}$$

In order to examine the influence of contact time on the adsorption process, adsorption experiments were performed at different time interivals while holding the other process parameters constant at their optimum values (pH, adsorbent dosage and MB initial concentration at 50 and 100 ppm). After a pre-set time interval, 3 mL aliquots were taken from the vials, centrifuged to separate adsorbent particles and analyzed for their residual MB concentration using the UV–vis spectrophotometer (UV-2600, Shimadzu, Japan). Every experiment was replicated thrice and mean values were adopted. The equilibrium was investigated using isotherm models and discussed in terms of its nonlinear equations; we proved our results by examining the statistical parameters *R*^2^, adjusted ${\bar{R}}^{2}$ and χ^2^ as shown in Equations (3)–(5):

(3) $$\chi^{2}=\sum {\left(qexp-qcal\right)}^{2}/qcal^{2}$$

(4) $$R^{2}=1-\sum {\left(q_{exp}-q_{cal}\right)}^{2}/1-\sum {\left(q_{exp}-q_{mean}\right)}^{2}$$

(5) $${\bar{R}}^{2}=1-\left(1-R^{2}\right)\left[\frac{n-1}{n-\left(k+1\right)}\right]$$

### 2.5. Quality Assurance and Results Reliability

A UV–vis spectrophotometer was used to measure the dye concentration remaining in the water. All glassware and plastic used in the experiments were washed with 5% HCl solution and immersed in bidistilled water. All reagents used in the experiments were of high analytical grade, and analytical precision in dye measurement was ensured by measuring MB solution standard with the UV–vis spectrophotometer to obtain a calibration curve with *R*^2^ = 0.999. Three standard solutions of MB were made to confirm the reliability of the results from the UV–vis spectrophotometer after every 15 samples. All experiments were performed in triplicate to ascertain their reproducibility, and the average concentration was determined by applying the mean and standard deviation (±SD).

## 3. Results and Discussion

### 3.1. Discussion of Characterization Results

**Figure 2:**
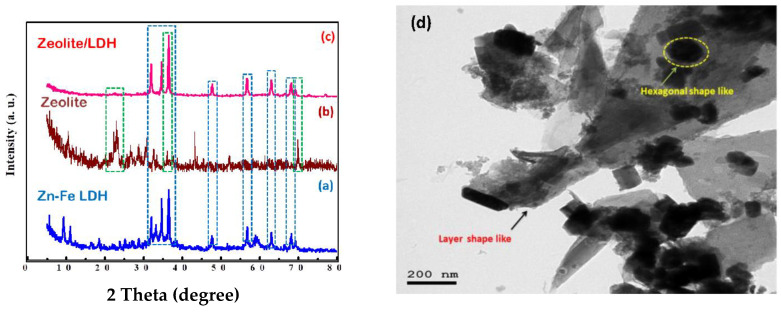
XRD patterns of the prepared samples (**a**–**c**); the HRTEM of Zn-Fe LDH (**d**).

The XRD [39] patterns of Zn–Fe LDH showed a crystalline layered phase. The basal spacing *d* value of the LDH phase, which represents the sum of the thickness of the brucite-like layer (0.414 nm) which is good agreement with that of the nitrate LDH materials with reference code (04-018-3495). The layered structure of the Zn-Fe LDH is ascertained by the presence of the main peaks of index (003), (006), (009), (012) and (018) at 2θ: 9.14°, 2534°, 31.86°, 47.70° and 56.90° (Figure 2a). It could be observed that the peaks were narrow, proving that the exchange of Zn and Fe into the crystalline Zn-Fe LDH structure were performed [39]. Since the peaks of (015) and (009) are higher intense than that for (003) which related to the formation of partly delaminated LDH is probable this coincides with the layered morphology in the HRTEM images for Zn–Fe LDH [40] (Figure 2d). The diffraction peaks 31.60°, 34.69°, 36.48°, 62.84°, 67.90°, and 69.44° had been indicated and indexed as the hexagonal phase of zinc oxide. The characteristic zeolite peaks appeared at 2θ values of 21.7°, 24°, 27.2°, and 30° (Figure 2b) [40,41]. In the composite, the diffraction peak of zeolite at 2θ = 27.2° was not observed; this is due to overlapping with the high-intensity peaks of Zn-Fe LDH. Also, the peak intensities of the composite sample were higher, indicating a higher degree of crystallization for the Zn-Fe LDH crystals in zeolite/LDH compared with that for pure Zn-Fe LDH [30,42]. Furthermore, some peaks shifted, e.g., 22.2 to 22.56°, 31.96 to 31.98, and 36.46 to 36.47, while the other peaks remained unchanged (Figure 2a–c). The crystallite sizes were 35.10, 40.21, and 50.35 nm for Zn-Fe LDH, zeolite, and zeolite/LDH, respectively, which confirms the formation of nanocomposite with a greater crystallite size. which agrees with the measured decreases in the BET surface area. The XRD pattern of the zeolite/LDH (Figure 2c) indicates that peaks representing the zeolite phase and LDH phase are present, indicating the successful and effective growth of LDH crystals on the zeolite partials. Also, the intensity of the diffraction peaks of zeolite/LDH increase and sharpen may be due to the dramatic increase of the crystallite size [43].

**Figure 3:**
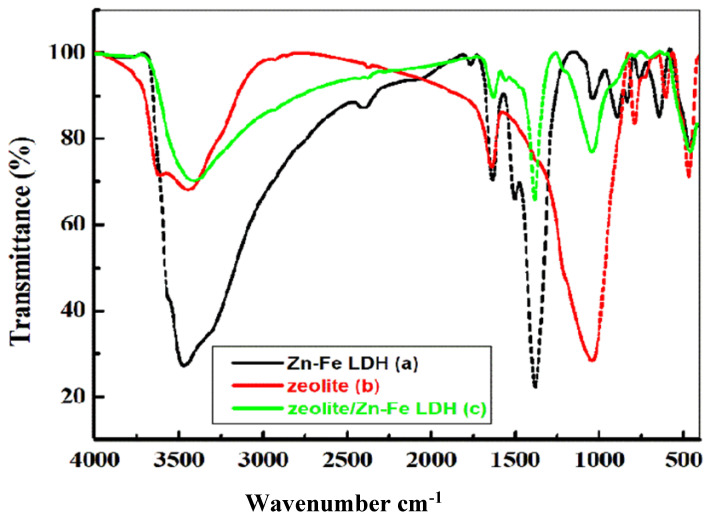
(**a**–**c**) FT-IR spectra patterns of Zn-Fe LDH, zeolite and zeolite/Zn-Fe LDH composite.

Figure 3a–c show the FT-IR spectra of the Zn-Fe LDH, zeolite, and zeolite/Zn-Fe LDH composite, respectively. Similar spectra were observed for the three materials. The FT-IR spectrum of Zn-Fe LDH (Figure 3a) presents a typical band at 1357 cm^−1^ confirming the presence of NO_3_^−1^ groups. It also shows a sharp peak at 1650 cm^−1^ and a broad peak at 3400 cm^−1^, which were attributed to the stretching mode of the OH- group of water molecules into the interlayer of LDH, and the broadness of such a band signals a variety of hydrogen bonds at the Zn-Fe LDH surface [16]. It is clear that water has different absorption bands in zeolite materials. The strong bands at 3400 and 1630 cm^−1^ were assigned to vibration of the bonds O–H-O and the bending mode of water [44]. The band at 437 cm^−1^ was assigned to the bending vibration of O–Al–O and O–Si–O bonds [30,44]. Bands in the region 500–800 cm^−1^ are probably due to pseudo-lattice vibrations and show the nature of the channel cations [45]. These spectra confirm the incorporation of zeolite into the Zn-Fe LDH structure. The bands appearing in the range of 1000–400 cm^−1^ are due to the stretching and bending modes of Zn–O and Fe–O bonds [35]. In the spectra of zeolite and zeolite/Zn-Fe LDH, the band at 3490 cm^−1^ was attributed to the Al–OH group on the surface of zeolite and the Si–OH group [30] (Figure 3b). The strong band at 1045 cm^−1^ is related to the *M*–O (*M* = Si or Al) stretching vibration [45] These spectra confirm the incorporation of zeolite into the Zn-Fe LDH structure.

**Figure 4:**
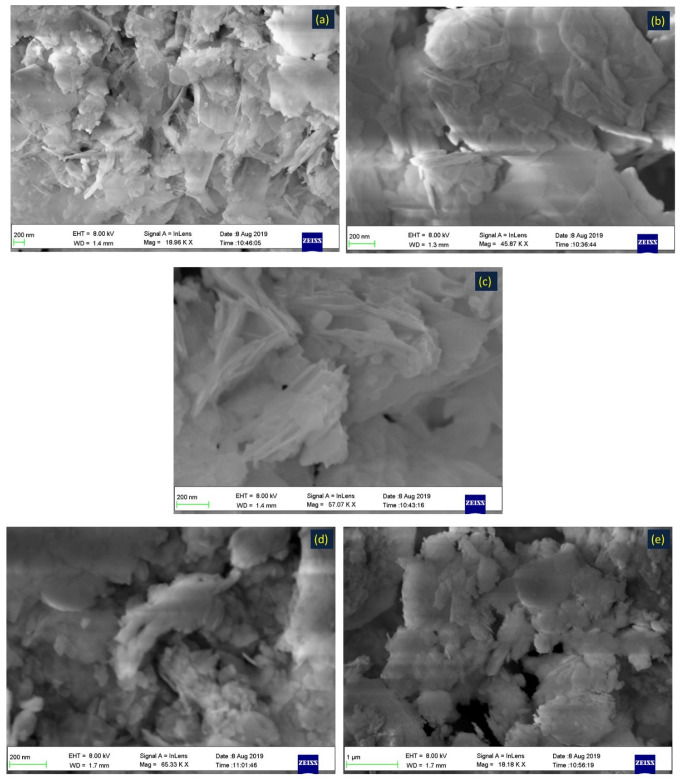

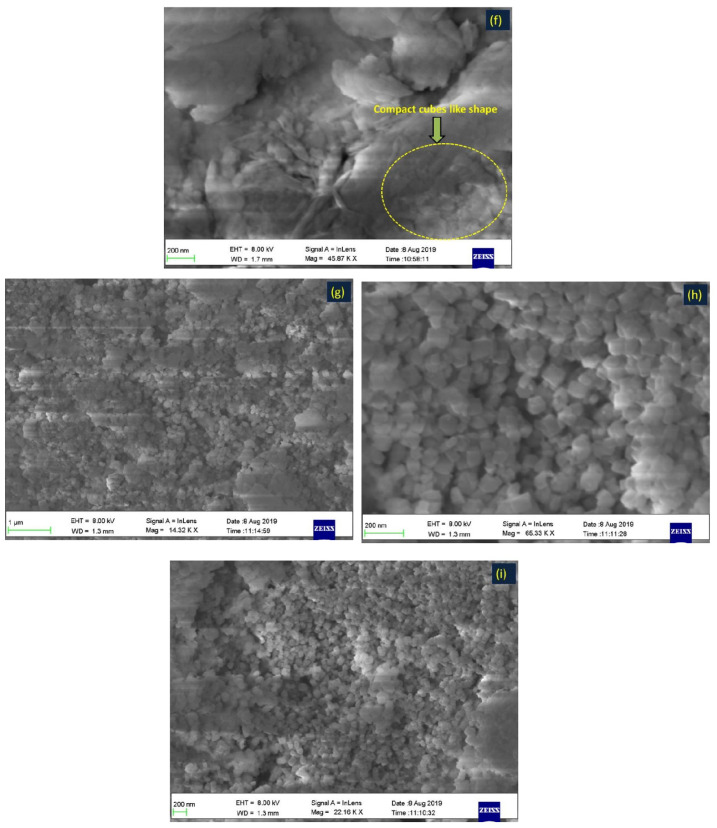
FESEM images of the prepared Zn-Fe LDH (**a**–**c**), zeolite (**d**–**f**) and zeolite/Zn-Fe LDH (**g**–**i**).

**Figure 5:**
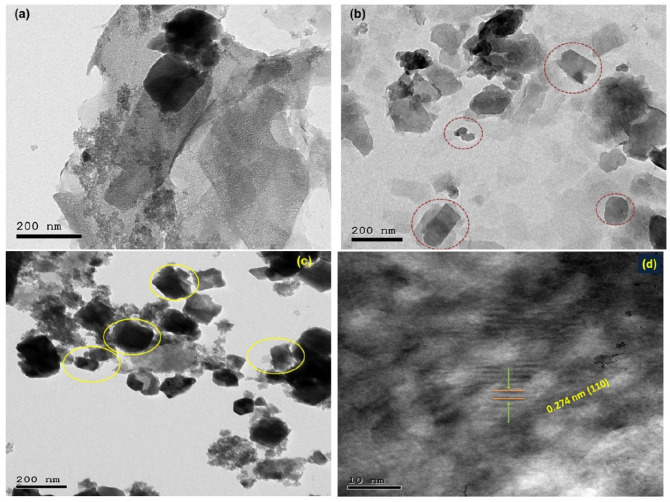
HRTEM images of the prepared Zn-Fe LDH (**a**), zeolite (**b**) and zeolite/Zn-Fe LDH (**c**) and in situ magnification for the Zn-Fe LDH (**d**).

The morphology of the synthesized materials was studied via FE-SEM (Figure 4). The layer and sheet Zn-Fe LDH nanostructures are shown in Figure 4a–c. The FE-SEM images of both Zn-Fe LDH and zeolite (Figure 4d–f) were layered structures, however zeolite/Zn-Fe LDH present well-defined cubic shapes. It is believed that the change in LDH morphology (Figure 4g–i) can be attributed to the zeolite acting as a structural directing agent by formation of complex structures that can be adsorbed on certain crystal planes of layer growth zones in certain directions. Figure 5a displays the HRTEM micrographs of Zn-Fe LDH which illustrates the layer-like shape and confirms the layered structure [14]. However, after synthesis of Zn-Fe LDH in the presence of zeolite (Figure 5b), we observed interesting images of a cube-like structure (Figure 5c). The reason for this behavior may be as a result to the interaction between the zeolite and the LDH metal ions during the slow addition of NaOH. In order to confirm our explanation, we investigated the morphology of zeolite to confirm the new morphology. It was found that the crystal particle structure with interplanar spacing is about 0.274 nm, which matched with the results obtained from the XRD data, indicating the layered structure of Zn-Fe LDH [46].

**Figure 6:**
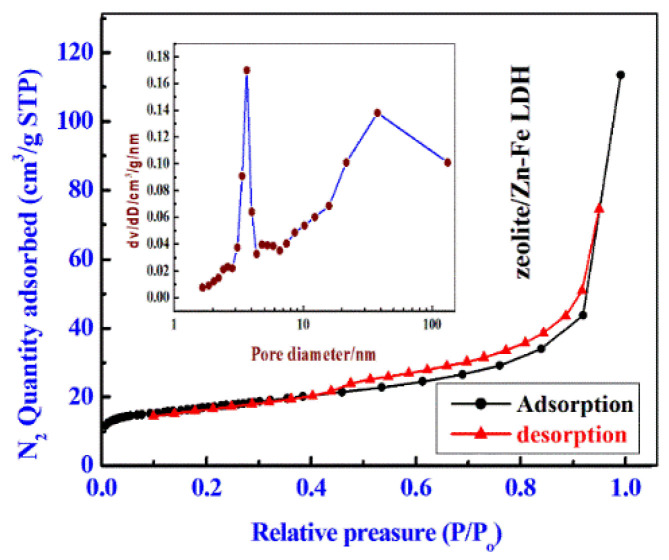
N_2_ adsorption/desorption isotherms zeolite/Zn-Fe LDH nanocomposite and the inset figure is BJH pore size distributions.

Figure 6 and Figure S2 show the N_2_ adsorption/desorption isotherms at 77 K on Zn-Fe LDH, zeolite, and zeolite/LDH composite to investigate their porosity and texture characters. All isotherms closely resemble type IV according to the International Union of Pure and Applied Chemistry (IUPAC) classification, which is characteristic of microporous and mesoporous materials. At a higher relative pressure (0.40 < P/Po < 0.95), the hysteresis loop in zeolite is larger than that of LDH, which suggests that zeolite contains a larger amount of mesopores and surface area than does LDH [44,47]. This was further ascertained by the BJH pore-size distribution plot (inset curves in Figure 6 and Figure S2) which obviously demonstrated the attendance of mesopores with an average size of approximately 3.6 nm for Zn-Fe LDH, 3.71 for zeolite and 3.33 for zeolite/Zn-Fe LDH. The specific surface areas of the Zn-Fe LDH, zeolite, and zeolite/LDH composite were 16.85, 59.83, and 55.94 m^2^/g, respectively. It seems that zeolite’s surface characteristics are predominant in the composite. The total pore volume of the zeolite (0.15 cm^3^/g) was higher than that of the LDH (0.07 cm^3^/g/g) or zeolite/LDH composite (0.018 cm^3^/g) (Table 3).

### 3.2. Discussion of Adsorption Studies

**Figure 7:**
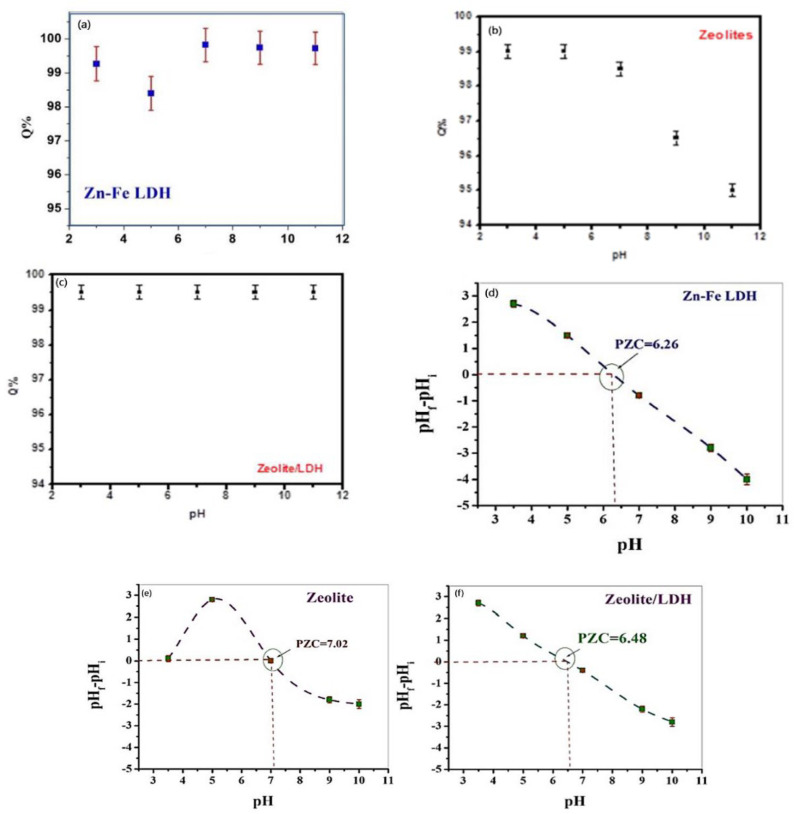
The effect of pH on the adsorption of MB using Zn-Fe LDH (**a**) zeolite (**b**), zeolite/LDH nanocomposite (**c**). and pH PZC values of Zn-Fe LDH (**d**) zeolite (**e**), zeolite/LDH nanocomposite (**f**) at different pH values.

The adsorption of MB molecules is strongly affected by the pH of the solution. The adsorption mechanisms of MB are surface complexation and exchange reaction. However, both of these reactions depend mainly on the solution pH. The results of MB adsorption onto zeolite showed that the removal rate decreased with increasing pH, although the removal percentage was high (95–100%) in the pH range from 3 to 11 (Figure 7).

These results can be explained as follows: The surface of an adsorbent is transformed from positively charged to negatively charged as the pH of the solution reaches the point of zero charge (pH_pzc_~7). At pH < pH_PZC_, the surfaces of the three prepared materials are positively charged due to the protonation process. Subsequently, the staking of MB molecules onto LDH is limited due the effect of coulombic repulsion. On the other hand, when the pH of the solution is >pH_PZC_, the adsorbents’ surfaces become negatively charged as a result of the deprotonation process, which promotes electrostatic attraction between the adsorbents and MB molecules. The variation in the charge of the adsorbent surface with the solution pH and pH_pzc_ is shown in Figure 7a. Figure 7d shows that the adsorbents were negatively charged within a wide pH range (pH > 6.26).

The processes of sedimentation and aggregation also vary with change in the solution pH, and maximum aggregation happens when the pH of the solution reaches pH_pzc_ [48]. Above pH_pzc_, the negativity of the surface charge increases as the pH increases, resulting in an increase in electrostatic repulsion forces and a decrease in the aggregation intensity. Over the pH range under study, the highest adsorptivity occurred at pH 7, where the aggregation reached its minimum level. There is competition between H^+^ and MB at the same active sorption sites of the adsorbent: the concentration of H^+^ decreases as the solution pH increases, and additional free sorption sites are thus available for MB adsorption. It can be concluded that pH 7 is the optimum pH for the adsorption of MB onto LDH.

In case of the zeolite under study, it seems that there were two points of zero charge (Figure 7e). Between these points (pH 3.5 and pH 7.02), the removal percentage was high (Figure 7b) due to the existence of negative charge favoring the adsorption of the cationic dye. Although there are plenty of H^+^ ions in the solution at lower pH, the negative charge of the anions in the inner surface of the zeolite is still able to attract more MB molecules. The results showed high removal efficiency of around 100% within a wide pH range, which indicates that the combination of zeolite and LDH in this study resulted in a composite with higher mechanical and chemical stability than either LDH or zeolite alone, in addition to combining their adsorptive power.

**Figure 8:**
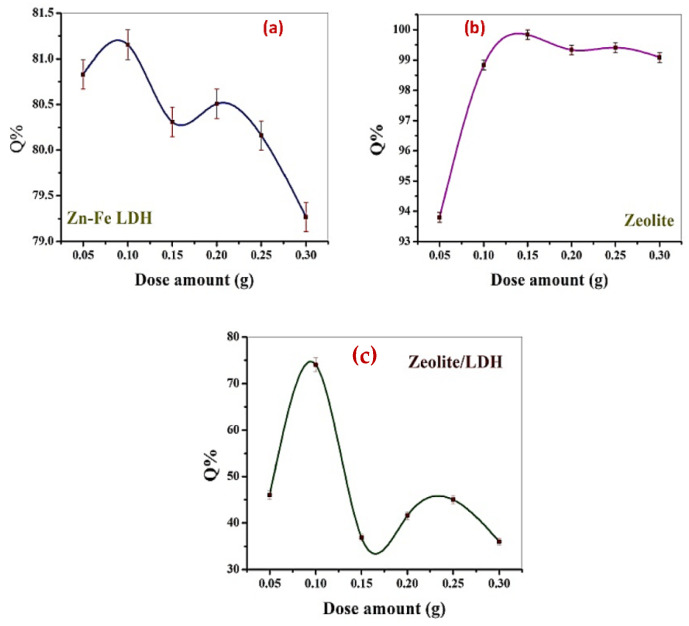
The effect of adsorbent dose on the adsorption of MB using Zn-Fe LDH (**a**) zeolite (**b**), and zeolite/LDH nanocomposite (**c**).

Figure 8 illustrates the influence of the adsorbent dose (0.05–0.30 g) on MB removal with 50 mg/L as C_o_ at pH = 7. The removal percentage increased with an increasing amount of zeolite, which is attributed to the presence of additional free sites in the higher amount (Figure 8a). This was in contrast to the other materials, where decreasing the Zn-Fe LDH (Figure 8b) and zeolite/LDH (Figure 8c) dosages increased their removal efficiencies. The aggregation of natural or synthetic clays often occurs at a high dosage of such materials. Subsequently, as the adsorbent dosages increased, the number of active sites decreased and the MB removal efficiency thus decreased. Hence, implementing a low dosage of the adsorbents is highly recommended. Additionally, the implementation of small dosages of nanoparticles is preferable in industry as it decreases the overall cost of the treatment process, while increasing the dosage results in negative environmental impacts and increases the cost of disposal [49,50]. The different trend in the removal percentage in the case of zeolite agreed with the previous results regarding pH, where the presence of plenty of negative charge in the inner surface of zeolite instead of its outer surface reduces the repulsion between the molecules; subsequently, its diffusion in the solution increases, resulting in increased absorptivity.

### 3.3. Adsorption Isotherms

Adsorption isotherms are necessary to understand the reaction between molecules in solid and liquid phases and also to estimate the *q_m_* values of the Zn-FeLDH, zeolite, and zeolite/LDH nanocomposite. Two-parameter models are commonly applied due to their simplicity and ease of fitting. The Langmuir, Freundlich, and Langmuir–Freundlich adsorption modelling isotherms were used to fit the experimental data using a nonlinear relationship. The Langmuir isotherm model (Equation (6)) was studied to assess the maximum adsorption capacity *q_m_* [43]:

(6) $$q_{e}=q_{m}k_{L}C_{e}/(1+k_{L}C_{e})$$

The Freundlich isotherm model, Equation (7) [51], assumes a heterogeneous adsorption surface:

(7) $$q_{e}=k_{f}C_{e}^{1/n}$$

The Langmuir-Freundlich isotherm is considering a mix of the above-mentioned isotherms (Equation (8)). It explains the adsorption energy at heterogeneous surfaces of sorbents via the following Equation (8) [47]:

(8) $$q_{e}=q_{MLF}{\left(K_{LF}C_{e}\right)}^{MLF}/{\left(K_{LF}C_{e}\right)}^{MLF}$$

As shown in Figure S3 and Table 4, the MB adsorption onto Zn-Fe LDH, zeolite, and zeolite/LDH nanocomposite was well fitted by the three models with the order: Langmuir > Freundlich > Langmuir–Freundlich. Based on the values of the correlation coefficient (*R*^2^), both the Langmuir and Freundlich models were optimal isotherm models with *R*^2^ close to unity (*R*^2^ = 0.99 for both models), adjusted correlation coefficient and the smallest Chi^2^ values. The results proved that using zeolite/LDH nanocomposite was more efficient for MB removal from a synthetic solution. The *q_e_* value of MB was up to 932.31 mg/g for the zeolite/LDH nanocomposite, greater than those for the zeolite (749.99 mg/g) and Zn-Fe LDH (37.58 mg/g).

The values of 1/*n*_F_ (Table 4) show that the LDH surface is more heterogeneous than that of the nanocomposite, while adsorption onto zeolite is cooperative. However, the maximum adsorption capacities for the composite according to the Langmuir model are much higher than those for both LDH and zeolite, while the specific surface area for zeolite is higher than that for the composite and much higher than that for LDH; this suggests that the internal surface area is not the controlling parameter for the adsorption of MB onto the prepared adsorbents and that the functional groups on the adsorbents play an important role in the process.

### 3.4. Adsorption Kinetics

**Figure 9:**
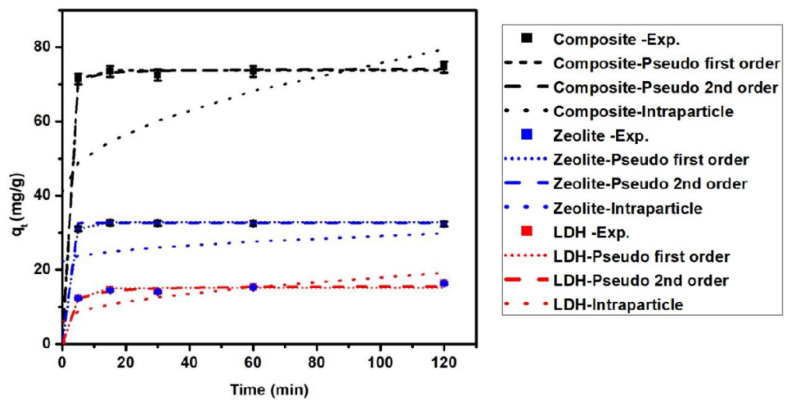
Comparison of adsorption kinetic models with experimental data of MB dye on LDH, zeolite and composite at concentrations 50 mg/L.

Studying the kinetics of adsorption for MB onto the prepared adsorbents is very important to determining the rate of MB removal and helps in predicting the removal mechanism. As presented in Figure 9, the removal rate was high in the first 5 min for LDH and zeolite/LDH, and then gradually increased until equilibrium at 15, 20, and 20 min for zeolite, LDH, and the nanocomposite, respectively. The long time taken to reach equilibrium may be due to the availability of adsorption through a chemisorption mechanism, taking more time. The adsorption kinetics of MB was studied by simulations of the experimental data with a pseudo-first-order model (Equation (9)), pseudo-second-order model (Equation (10)), and intraparticle diffusion model (Equation (11)) [52] as follows [47]:

(9) $$q_{t}=q_{e}\left(1-e^{-K_{1}t}\right)$$

(10) $$q_{t}=\frac{K_{2}q_{e}^{2}t}{1+K_{2}q_{e}t}$$

*qt* = *K_ip_√t* + *C_ip_* (11)

**Figure 10:**
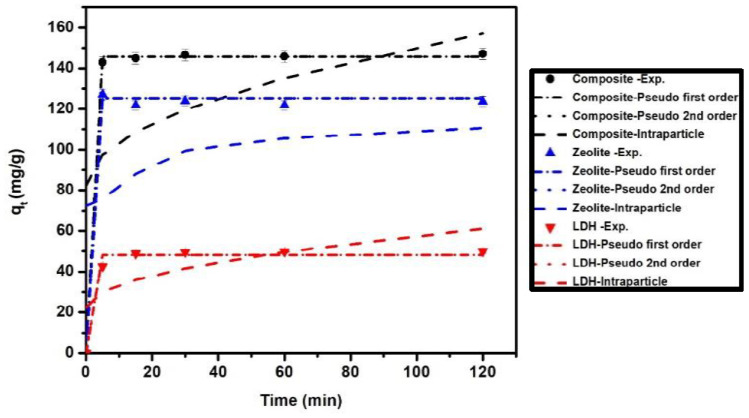
Comparison of adsorption kinetics models with experimental data of MB dye on LDH, zeolite and composite at concentrations 100 mg/L.

The parameters of the simulated models (Table 5) showed that the correlation coefficients (*R*^2^) of the pseudo-second-order and pseudo-first-order models were higher than that of the intraparticle diffusion model. The adsorption capacities (*q_t_*) resulting from simulation by the pseudo-first-order and pseudo-second-order models were very close to the experimental values Figure 9 and Figure 10, in contrast to the intraparticle diffusion model. This indicates that the functional groups on zeolite, LDH, and the nanocomposite play a significant role in the adsorption mechanism of MB onto their surfaces

To study the mechanism of adsorption of MB on the LDH surface a representative example can be investigated using FT-IR spectra. The FT-IR spectrum of the Zn–Fe LDH, after the adsorption of MB on Zn–Fe LDH was confirmed through FT-IR analysis (Figure S4) as a representative example. We observe a small intense peak at 1618 cm^−1^ consistent with the C–C vibration band of the benzene ring related to the MB chemical structure. The peaks at 1138 and 1327 cm^−1^ were related to stretching vibrations of C–C and C–N, respectively. C–H stretching vibration peaks of the benzene ring were located at 1030 and 837 cm^−1^. Hence, we can conclude that MB is adsorbed to the surface of Zn-Fe LDH. Also, the basal spacing of the (003) plane decreased from 0.414 nm in the case of LDH to 0.693 in LDH/MB, which revealed a high effective penetration of MB into LDH interlayers This increasing may refer to one of the following reasons: the anion exchange of nitrate molecules, rearrangement of Zn-Fe LDH ions, and removal of water molecules or the adsorption of dye molecules on the surface of LDH via hydrogen-bonding. Also, the porous part of the prepared materials have important role in the MB adsorption process and attributed mainly to the size of the methylene blue molecule, which is 0.84 nm [1,53], since each molecule of this contaminant interacts with a single active site present on the surface of the prepared materials (Table 3).

## 4. Conclusions

In the present work, zeolite, Zn-Fe LDH, and zeolite/LDH composite absorbents were successfully synthesized and characterized by the XRD, FTIR, SEM, BET surface area, XRF and TEM techniques. The zeolite, Zn-Fe LDH, and zeolite/LDH composite were then used for MB removal from water. The adsorption capacity for MB removal was 932.31 mg/g for the zeolite/LDH composite, greater than those of zeolite (749.99 mg/g) and Zn-Fe LDH (37.58 mg/g). The adsorption data were fitted by three nonlinear isotherm models, which showed that the adsorption process is controlled kinetically by a pseudo-second-order model.

## Figures and Tables

**Table 1:** Comparison of adsorption capacity ($q_{m}$) for different adsorbents for removing of MB.

Adsorbent	Adsorption Capacity	Ref.
MgAl-LDH/Biochar composites	406.47 mg/g	[32]
ZIF-67@CoAl-LDH composites	57.14 mg/g	[33]
Zn/Al LDH/Rice Husk Biochar	62.39mg/g	
CoZnAl-LDH/GO nanocomposite	169.49 mg/g	[34]
Ca/Al LDH/biochar composites	32.535 mg/g	[35]
LDH	37.58 mg/g	This study
zeolite	749.99 mg/g	This study
zeolite/LDH composite	932.31mg/g	This study

**Table 2:** Conditions of the ball milling process for the preparation of Zeolite.

Process Condition	Description
Balls diameters	Range from 1.5 to 1.8 cm
Vessel diameter	7.5 cm
Materials of vessel	Stain steel
Materials of used balls	Porcelain
Ball/natural-zeolite mass ratio	10:1
Speed	200 rpm
Time	10 h

**Table 3:** The results of BET analyses for the synthesized samples; PV and APD are pore volume and average pore diameters.

Sample	Surface Area (m^2^/g)	PV (cm3/g)	ADP (nm)
Zn-Fe LDH	16.85	0.07	3.6
zeolite	59.83	0.15	3.71
zeolite/Zn-Fe LDH nanocomposite	55.94	0.018	3.33

**Table 4:** The parameters of the adsorption models for MB sorption onto Zn-Fe LDH, zeolite, and nanocomposite.

Isotherm Models	Parameter	Values	*R* ^2^	${\bar{R}}^{2}$	χ^2^
Zn-Fe LDH					
Langmuir	*q_max_* (mg/g)	37.58	0.89	0.74	0.002521
	*K*_ad_ (L/mg)	0.055			
Freundlich	*K*_f_ (mg/g)	4.38	0.84	0.70	0.003877
	1/*n*_F_ (-)	0.48			
Langmuir-Freundlich	*q_max_* (mg/g)	51.56	0.86	0.86	0.003388
	*K*_LF_ (L/mg)	0.027			
	β*_LF_* (-)	0.81			
zeolite					
Langmuir	*q_max_* (mg/g)	749.99	0.95	0.97	0.00272
	*K*_ad_ (L/mg)	0.0069			
Freundlich	*K*_f_ (mg/g)	3.50	0.96	0.80	0.00629
	1/*n*_F_ (-)	1.02			
Langmuir-Freundlich	*q_max_* (mg/g)	401.85	0.93	0.93	0.010704
	*K*_LF_ (L/mg)	0.023			
	β*_LF_* (-)	1.37			
zeolite/LDH composite					
Langmuir	*q_max_* (mg/g)	932.31	0.99	0.83	0.00146
	*K*_ad_ (L/mg)	0.002			
Freundlich	*K*_f_ (mg/g)	3.33	0.99	0.83	0.000815
	1/*n*_F_ (-)	0.83			
Langmuir-Freundlich	*q_max_* (mg/g)	433.91	0. 88	0.88	0.001097
	*K*_LF_ (L/mg)	0.008			
	β*_LF_* (-)	0.83			

**Table 5:** Kinetic parameters for the pseudo-first-order, pseudo-second-order, and intraparticle diffusion models of MB adsorption onto the LDH, zeolite, and zeolite/LDH composite.

Model	LDH		Zeolite		Composite	
Pseudo-first-order	C_o_, 50 mg/L	C_o_, 100 mg/L	C_o_, 50 mg/L	C_o_, 100 mg/L	C_o_, 50 mg/L	C_o_, 100 mg/L
*k*_1_ (min^−1^)	0.336	97.4	0.58	108.8	0.69	140.11
*q_t,cal_* (mg/g)	15.11	48.15	32.79	125.18	73.77	145.68
*R* ^2^	0.98	0.98	0.99	0.99	0.99	0.99
Pseudo-second-order						
*k*_2_ (g/mg min)	0.043	0.021	0.101	4.92	0.067	0.047
*q_e,cal_* (mg/g)	15.77	50.8	32.57	125.19	74.17	146.68
*R* ^2^	0.99	0.99	0.99	0.99	0.99	0.99
Intraparticle diffusion						
*K_ip_* (mg/g min ^(1/2)^)	1.195	3.54	0.69	2.67	3.51	6.837
*C_ip_* (mg/g)	6.07	22.2	22.169	85.27	40.94	82.18
*R* ^2^	0.59	0.49	0.25	0.25	0.37	0.35

## Data Availability

The data presented in this study are available on request from the corresponding author.

## Supplementary Materials

The following are available online at https://www.mdpi.com/article/10.3390/nano11123315/s1, Figure S1: XRF analysis of the Zeolite sample, Figure S2: N2 adsorption/desorption isotherms zeolite, Zn-Fe LDH nanocomposite and the inset figures is BJH pore size distributions, Figure S3: Experimental data for MB adsorption on Zn-Fe LDH (a) and MB adsorption on Zeolite (b) MB adsorption on Zeolite/LDH nanocomposite (c) fitted by the nonlinear isotherm models. The error bar for standard deviation reflects the replicate experiments, Figure S4: FTIR spectra for adsorption of MB on the LDH surface.

## Abbreviations

LDH	Layer double hydroxide
C_o_	The initial concentration
C_t_	The dye concentration (mg/L)
V	The volume of the dye solution (L)
W	The mass of the sorbent (g)
q_e_	Equilibrium adsorption capacity of adsorbent (mg/g)
C_e_	Equilibrium concentration of the adsorbate (mg/L)
Q%	Removal percent
χ ^2^	Chi square
q_exp_	Experimental adsorption capacity of adsorbent at equilibrium (mg/g)
q_cal_	Calculated adsorption capacity of adsorbent at equilibrium (mg/g)
$R^{2}$	Determination coefficient
${\bar{R}}^{2}$	Adjusted correlation coefficient
K_f_	Freundlich constant (L/g)
*n*	The sample size
*k*	The number of independent variables in the regression equation
1/n_f_	Freundlich adsorption intensity
*K*_LF_	Equilibrium constant for heterogeneous solid
k_1_	Rate constant of pseudo first-order model (1/min)
k_2_	Rate constant of pseudo second-order model (g/mg min)
k_ip_	Measure of diffusion coefficient (mg g^−1^ min^−1(1/2)^)
C_ip_	Intraparticle diffusion constant mg/g
