# Supplementary material for: LDH Nanocubes Synthesized with Zeolite Templates and Their High Performance as Adsorbents
Source: Nanomaterials (Basel). 2021 Dec 7;11(12):3315. doi: 10.3390/nano11123315 (PMC8708268; doi:10.3390/nano11123315)
Supplement: Supplementary file 1 [file nanomaterials-11-03315-s001.zip › nanomaterials-1421895-supplementary.pdf]

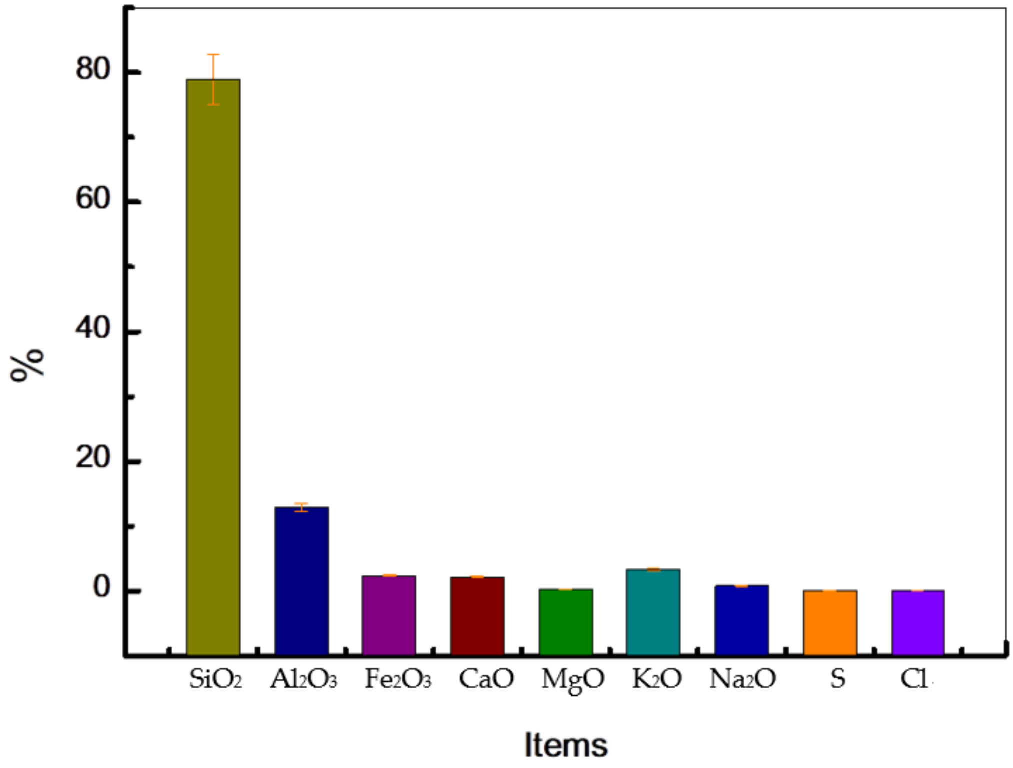

Figure S1. XRF analysis of the Zeolite sample.

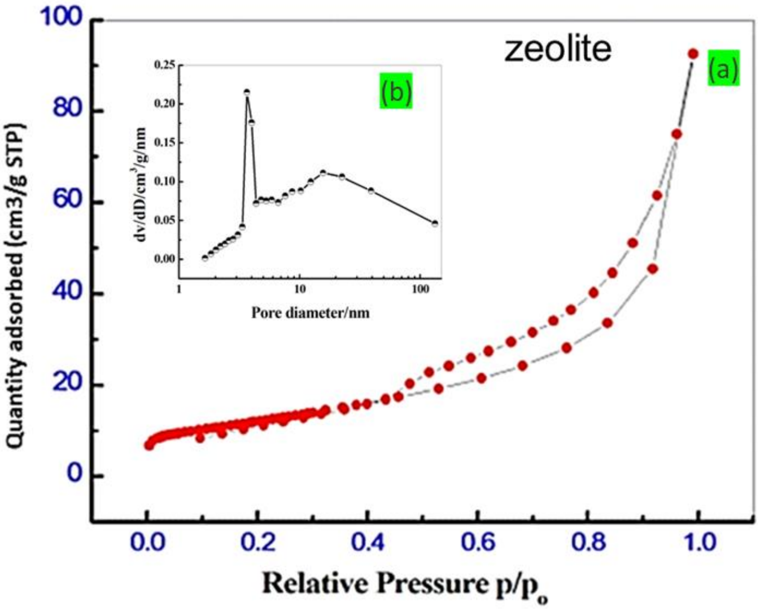

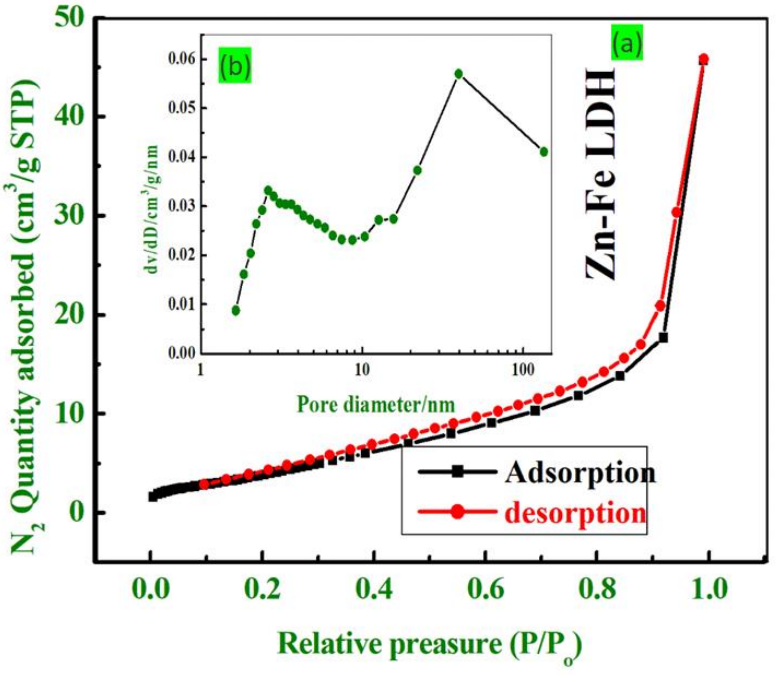

Figure S2. N_2 adsorption/desorption isotherms zeolite, Zn-Fe LDH (a) and the inset figures is BJH pore size distributions (b) for zeolite and Zn-Fe LDH.

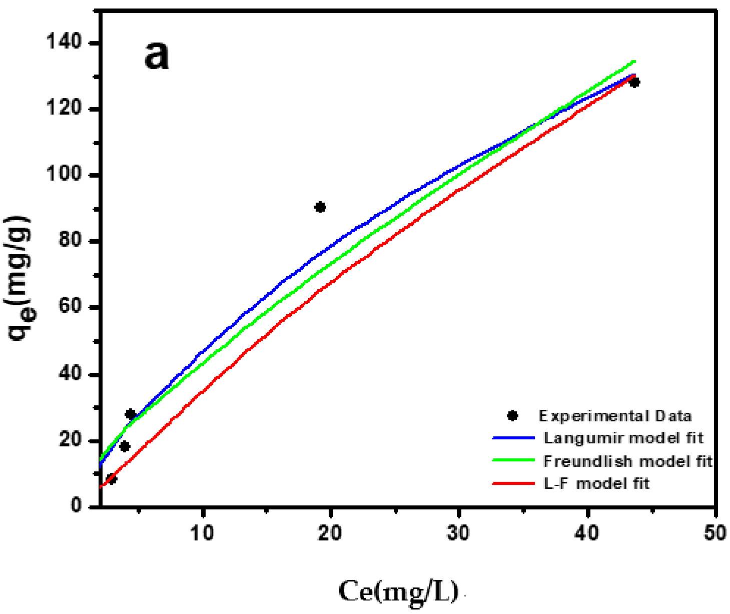

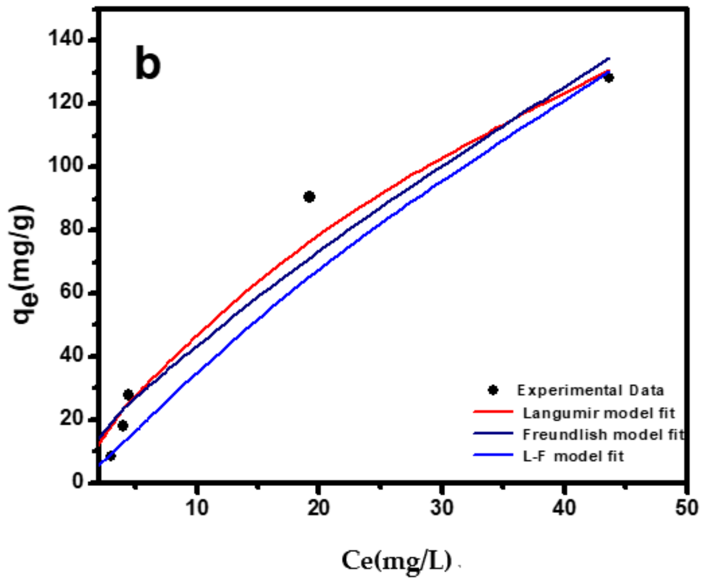

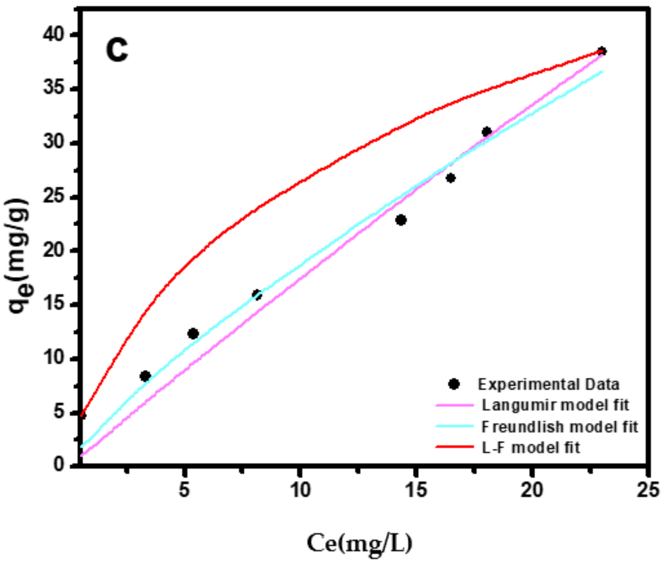

Figure S3. Experimental data for MB adsorption on Zn-Fe LDH (a) and MB adsorption on Zeolite (b) MB adsorption on Zeolite/LDH nanocomposite (c) fitted by the nonlinear isotherm models. The error bar for standard deviation reflects the replicate experiments**.**

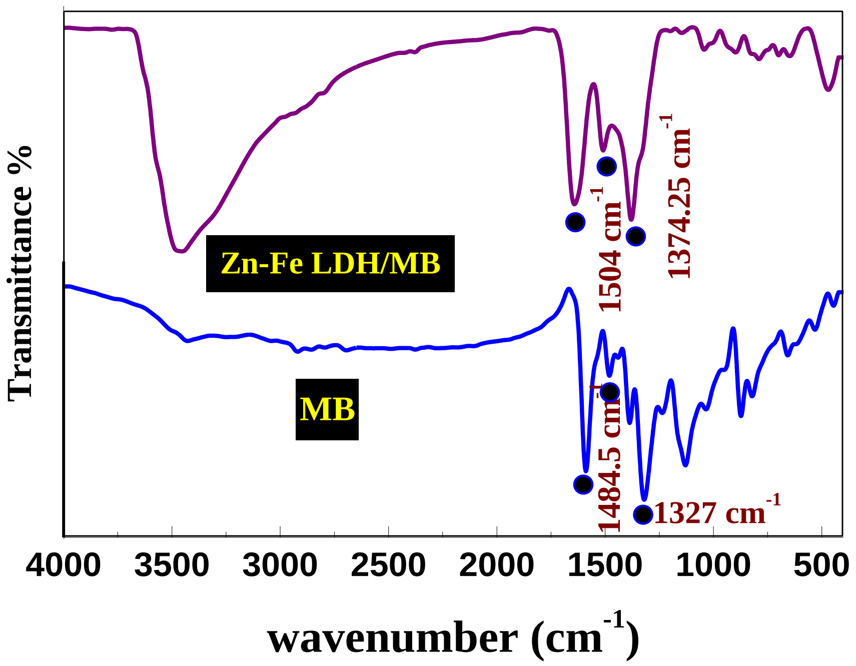

Figure S4. FTIR spectra for adsorption of MB on the LDH surface.
